# Chromogranin A: From Laboratory to Clinical Aspects of Patients with Neuroendocrine Tumors

**DOI:** 10.1155/2018/8126087

**Published:** 2018-07-02

**Authors:** Paola Di Giacinto, Francesca Rota, Laura Rizza, Davide Campana, Andrea Isidori, Andrea Lania, Andrea Lenzi, Paolo Zuppi, Roberto Baldelli

**Affiliations:** ^1^Endocrinological Oncology, Service of Endocrinology, A.O. San Camillo-Forlanini, Rome, Italy; ^2^Department of Medical and Surgical Sciences, S. Orsola-Malpighi University Hospital, Bologna, Italy; ^3^Department of Experimental Medicine, Sapienza University of Rome, Rome, Italy; ^4^Department of Endocrinology, Humanitas Clinical and Research Center, Humanitas University, Milan, Italy

## Abstract

*Background*. Neuroendocrine tumors (NETs) are characterized by having behavior and prognosis that depend upon tumor histology, primary site, staging, and proliferative index. The symptoms associated with carcinoid syndrome and vasoactive intestinal peptide tumors are treated with octreotide acetate. The PROMID trial assesses the effect of octreotide LAR on the tumor growth in patients with well-differentiated metastatic midgut NETs. The CLARINET trial evaluates the effects of lanreotide in patients with nonfunctional, well-, or moderately differentiated metastatic enteropancreatic NETs. Everolimus has been approved for the treatment of advanced pancreatic NETs (pNETs) based on positive PFS effects, obtained in the treated group. Sunitinib is approved for the treatment of patients with progressive gastrointestinal stromal tumor or intolerance to imatinib, because a randomized study demonstrated that it improves PFS and overall survival in patients with advanced well-differentiated pNETs. In a phase II trial, pasireotide shows efficacy and tolerability in the treatment of patients with advanced NETs, whose symptoms of carcinoid syndrome were resistant to octreotide LAR. An open-label, phase II trial assesses the clinical activity of long-acting repeatable pasireotide in treatment-naive patients with metastatic grade 1 or 2 NETs. Even if the growth of the neoplasm was significantly inhibited, it is still unclear whether its antiproliferative action is greater than that of octreotide and lanreotide. Because new therapeutic options are needed to counter the natural behavior of neuroendocrine tumors, it would also be useful to have a biochemical marker that can be addressed better in the management of these patients. Chromogranin A is currently the most useful biomarker to establish diagnosis and has some utility in predicting disease recurrence, outcome, and efficacy of therapy.

## 1. Background

Neuroendocrine tumors (NETs) originate from neuroendocrine cells of the diffuse endocrine system.

They are considered rare neoplasms, though their incidence and prevalence has increased in the last ten years [1].

According to the 2010 WHO classification [2], neuroendocrine neoplasms (NENs) are classified in neuroendocrine tumors (NETs), neuroendocrine carcinomas (NECs) of small or large cells, and mixed adenoneuroendocrine carcinomas (MANECs), showing more than 30% of neuroendocrine cells and nonendocrine components.

Based on the proliferative activity, assessed by mitotic count and/or the Ki-67 index, NETs are divided into NET-G1 (mitotic count < 2 per 10 high-power fields (HPFs) and/or Ki-67 ≤ 2%) and NET-G2 (mitotic count 2–20 per 10 HPFs and/or Ki-67 3 to 20%).

All NECs are considered G3 (mitotic count > 20 per 10 HPFs and/or Ki-67 > 20).

Furthermore, NENs distinguish between well-differentiated neuroendocrine tumors (NET-G1 and -G2) and poorly differentiated neuroendocrine carcinomas (NEC-G3).

It is now known that the G3 NEN is a very heterogeneous category of tumors. Morphological differentiation and Ki-67 are able to influence the prognosis of G3 groups, and therefore, a distinction between a well-differentiated G3 NET and a poorly differentiated G3 NEC would be useful.

Particularly, Heetfeld et al. proposed a G3 NET category defining NEN with well-differentiated features and Ki-67 < 55%, representing an intermediate category between G2 NETs and NECs [3]. Considering the prognostic role, the morphological categories that are taking place are well-differentiated G3 NETs with Ki-67 comprised between 20% and 55% and poorly differentiated neuroendocrine carcinomas (G3 NECs) with Ki-67 > 55%, especially in the GEP-NEN group.

The most common sites of NETs are the lung, stomach, appendix, cecum, duodenum, pancreas, jejunum/ileum, colon, and rectum [4].

NETs originating in the gastrointestinal tract and pancreas are known as gastroenteropancreatic neuroendocrine tumors (GEP-NETs) and are more frequent in gastric fundus-corpus, proximal duodenum, Vater's papilla, pancreas, tip of the appendix, terminal ileum, and lower rectum.

GEP-NETs constitute about 2% of all neoplasms; they are slow-growing and indolent neoplasms [5, 6].

Gastrointestinal NETs are rising [7]; in fact there was a 7-fold increase in the last 35 years, having available advanced endoscopic and radiological imaging [8–10].

They account for about 50–70% of all NETs [4], and they cause several pathological conditions since they can secrete biologically active substances leading to the development of characteristic clinical syndromes (functioning tumors).

However, the majority of GEP-NETs are not symptomatic and present with metastases or symptoms of mass effect (nonfunctioning tumors).

As GEP-NETs are a heterogeneous group of neoplasms, they are difficult to diagnose. Many patients may present with metastatic disease at initial diagnosis, thus having delays in receiving effective treatment with serious impact on prognosis and survival [4].

That is why it would be necessary to identify appropriate biomarkers to improve the management of these patients.

The ideal biomarker for GEP-NETs would be pretty specific for GEP-NETs, easily measured, able to identify both functioning and nonfunctioning tumors, and should be essential for treatment monitoring and disease prognosis.

Determination of serum chromogranin A (Cg A) is the most commonly used test [11].

Elevated serum Cg A is observed in patients with different types of endocrine tumors including carcinoid tumors of the stomach, lung, intestine, prostate, and liver; pheochromocytomas; parathyroid carcinomas; medullary thyroid carcinomas; anterior pituitary tumors; pancreatico-duodenal tumors; neural tumors; and small cell lung cancers (SCLCs) [12–36].

Interestingly, also certain nonneuroendocrine tumors, such as non-small-cell lung cancer (NSCLC), prostate cancer, and breast cancer, may undergo neuroendocrine differentiation and present focal expression of Cg A [12].

In addition, it is a component of dense-core synaptic granules in many areas of the central nervous system [13, 14].

## 2. Granin Family and Biological Activities

Chromogranin A is a 439-residue-long glycoprotein [15, 16], a member of a larger family of soluble secretory proteins (granin proteins) that includes also chromogranin B (CgB), secretogranin (Sg) II (CgC), Sg III (1B1075), Sg IV (HISL-19), Sg V (7B2) and Sg VI (NESP55), Sg VII (VGF), and Sg VIII (proSAAS) [12], localized in secretory granules of neuroendocrine cells, neurons, and the adrenal medulla [17].

These proteins enter into the rough endoplasmic cisternae, are transported to the trans-Golgi network (TGN), and then are targeted into dense-core secretory granules (DCGs), known as large dense-core vesicles (LDCVs), or into chromaffin granules (CGs), in the adrenal medulla.

In humans, Cg A is the precursor to several functional peptides including vasostatin-1 (VST I: hCgA1–76), vasostatin-2 (VST II: hCgA1–115), pancreastatin (PST: hCgA357–428), catestatin (CST: hCgA352–372), and parastatin (PARA: pCgA347–419), negatively modulating the neuroendocrine function [18].

Cg A is also a granulogenic protein and, when it is overexpressed in fibroblasts, induces granule-like structures with a dense core, releasing their contents.

This hydrophilic glycoprotein is encoded by the *CHGA* gene, located in chromosome 14q32.12.

The N-terminal domain of Cg A is responsible for directing Cg A into the secretory granules [19, 20] and for binding to Sg III, the receptor for Cg A, in the presence of calcium at the level of the pituitary cells and pancreatic beta cells and in neuroendocrine cells [21, 22].

At the moment, a universal diagnostic technique is unavailable and the assessment of plasma concentrations of Cg A can be achieved by several commercial kits, such us radioimmunoassay (RIA), enzyme-linked immunosorbent assay (ELISA), immunoradiometric assay (IRMA), Western blotting, immunofluorescence microscopy, immunohistochemistry, and by recent immunofluorescent TRACE assay [23].

The first radioimmunoassay for measurement of chromogranin A was introduced in 1986 [24], and the kit used antibodies against human chromogranin A.

The IRMA assay makes use of two monoclonal antibodies, binding the unprocessed central domain of chromogranin A.

Conversely, the ELISA method is based on two polyclonal rabbit antibodies raised against a 23 kDa carboxyl-terminal domain of human chromogranin A.

Moreover, because it may be measured from serum or plasma, Glinicki et al. report that plasma concentrations of Cg A would be higher than those in serum [25].

Granins are precursor proteins that can give rise to a large number of small bioactive peptides, involved in different biological functions (Table 1).

The N-terminal Cg A fragment, localized within secretory granules, where insulin is also present, impedes insulin secretion from islet cells in response to glucose by blocking calcium influx. Pancreastatin induces glycogenolysis, inhibits the glucose-induced insulin release through an effect on G-protein and calcium-mediated exocytosis [26, 27], and also stimulates insulin-dependent lipogenesis but inhibits leptin secretion [28, 29].

Sympathetic tone regulates basal plasma levels of Cg A [30, 32] and controls both vascular tone and cardiac contractility [32].

In effect, plasma levels of CG A and PST (357–428) are increased in patients with essential hypertension [32–34]; conversely, plasma CST (352–372) is diminished in such patients [35]. Furthermore, this peptide and VST I (1–76) are involved in vasculogenesis [36], contributing to generate also a state of hypertension.

CST is considered the most potent inhibitor of nicotine-evoked catecholamine secretion [37, 38], and it is also a potent vasodilator [39, 40].

The granin family is involved in the regulation of inflammatory response: catestatin stimulates chemotaxis of human peripheral blood monocytes [41], with an effect comparable to vascular endothelial growth factor (VEGF) [36].

On the contrary, the Cg A-derived peptide VST I prevents VEGF-mediated chemotaxis [42].

Elevated serum calcium concentration and Cg A-derived peptides act on the calcium sensors present on the cell membrane [43]. VST I and Cg A peptides, located in parathyroid granules, are cosecreted when the serum calcium is low, making an inhibitory effect on parathormone secretion [44].

In the same way, cosecretion of parathormone and Cg A is inhibited by parastatin [45].

### 2.1. Causes of Elevated Chromogranin Unrelated to Neuroendocrine Tumor

In clinical practice, there are several nononcological conditions characterized by an increase in Cg A serum concentration, considering potential diagnostic pitfalls.

The use of proton pump inhibitors and other acid-suppressive medications causes elevated Cg A levels, as well as in the presence of atrophic gastritis.

For this reason, the determination of blood chromogranin A levels should be made after a drug-free period of at least 7 days.

Histamine type-2 receptor antagonists may increase the marker concentration. It is recommended to discontinue these drugs at least 24 h before the test.

Other benign diseases of the alimentary tract affecting the concentration of chromogranin A are pancreatitis, chronic hepatitis, liver cirrhosis, irritable bowel, and inflammatory bowel diseases.

Other nononcological conditions and oncological diseases inducing elevations of Cg A are listed in Table 2.

### 2.2. Chromogranin a and NET

The National Institutes of Health (NIH) has classified biomarkers into three categories [46]:

- “Type 0”: markers of the natural history of a disease correlate longitudinally with known clinical indices (symptoms) over the full range of a disease state
- “Type I”: capture the effects of an intervention in accordance with the mechanism of drug action, even though the mechanism might not be known to be associated with clinical outcome reflects interventional effects
- “Type II”: considered as surrogate end points because a change in the marker predicts clinical benefit

Optimally, a biomarker should be found uniquely in the malignant tissue of interest and generate a positive signal that can be measured without confounding “noise” from normal tissues or other nonmalignant abnormalities [47].

#### 2.2.1. Type 0 Biomarkers

Previous studies have demonstrated elevated circulating Cg A levels in serum or plasma of patients with neuroendocrine tumors. Chromogranin A historically has sensitivities that range between 60 and 100% with specificities of 70–100% depending on the NET type [48].

*(1) Specificity*. The principal limitation of Cg A evaluation is the low specificity (10–35% specificity).

As previously described, Cg A is elevated in other nonneuroendocrine neoplasia such as breast cancer, hepatocellular carcinoma, pancreatic cancer, colorectal cancer, ovarian cancer [49], and prostate cancer [50].

Cg A elevation may be caused by several gastroenteropancreatic diseases: autoimmune chronic atrophic gastritis [51], inflammatory bowel disease [52], irritable bowel syndrome [53], chronic hepatitis, and liver cirrhosis [54].

One of the commonest causes of spuriously elevated Cg A levels is the proton pump inhibitor (PPI) administration [55]. As reported by Mosli et al., short-term PPI use results in a significant increase of Cg A in serum and plasma, an effect that is largely independent of the assay used [56]. For this reason, PPI needs to be discontinued for 2 weeks to fully eliminate its effect on Cg A.

Moreover, there are renal diseases increasing Cg A levels, for example, impaired kidney function with reduced renal clearance [57]. Heart failure, classified according to the New York Heart Association (NYHA) scale, correlates with elevated Cg A [58]. And not for nothing, the concentration of chromogranin A relates with levels of brain natriuretic peptide (BNP) [59, 60]. There is a high concentration of Cg A also in cases of acute coronary syndromes, and if marker levels are increased significantly, the patient's prognosis worsens [61]. Elevated Cg A also occurs in case of untreated hypertension, and as the severity of the clinical condition correlates with increased adrenergic activity, levels of the marker are more or less increased.

There is a high concentration of Cg A in several inflammatory diseases, such as rheumatoid arthritis [62] and systemic lupus erythematosus.

Endocrine disorders associated with elevated chromogranin are pheochromocytoma, hypercortisolemia, hyperthyroidism, medullary thyroid cancer, hyperparathyroidism, and pituitary tumors (except prolactinomas).

Other factors affecting Cg A levels are strenuous exercise and food intake before the test.

*(2) Cg A and Tumor Localization*. Some studies have reported an association between Cg A and tumor location. Nobels et al. showed elevated Cg A in 100% of cases of gastrinomas, in 80% of small bowel NETs, and in 69% of nonfunctioning pNETs (NF-pNETs) and in the other endocrine tumors [63]. In GEP-NETs, biomarker concentration has been measured 100 times above the upper normal limit: in detail, the highest Cg A levels were 200 times the normal upper limit in ileal NETs and 150 times the normal upper limit in GEP-NETs associated with multiple endocrine neoplasia type 1 (MEN-1) [18].

Conversely, lower Cg A levels were noted in gastric type I, ranging from 2 to 4 times normal, whereas type II and III gastric ECLomas had intermediate values of the marker [18].

The determination of Cg A plasma levels does not play a key role in diagnosis of patients with colorectal NEN, because Cg A values in colorectal NENs are not significantly elevated [64].

Furthermore, these authors are saying that there is no correlation between elevated plasma Cg A levels and immunohistochemical positivity in the NEN tissue sections of the colon and rectum.

In the case of lung NETs, such as primary localization, the Cg A values were detected significantly lower than the levels of patients with GEP tumors [65].

*(3) Cg A and Tumor Differentiation/Tumor Burden*. The concentration of Cg A is correlated with the degree of neuroendocrine differentiation and tumor burden, especially with liver cancer burden [66].

Indeed, the diagnostic accuracy of Cg A measurement for GEP-NET is higher for well-differentiated tumors versus poorly differentiated disease [67].

Furthermore, the concentration of Cg A is higher in tumors with intense secretory activity, such as small bowel NETs causing carcinoid syndrome [68] and in disseminated rather than localized neoplastic disease. Several studies document how patients with multiple liver metastatic diseases show higher levels of Cg A than patients with limited neoplastic disease [65, 69].

Cg A concentration must also be elevated in the case of nonsecreting pancreatic NETs (NF-pNETs) with liver metastases at diagnosis.

In a retrospective single-center clinical study, conducted by Yang et al., Cg A values were statistically correlated with the well-differentiated NF-pNET in the presence of hepatic metastatic disease, even if Cg A levels were not statistically significant between well-differentiated and poorly differentiated pNETs [70].

Moreover, the authors emphasize how the Cg A concentration is connected with the degree of tumor load extension. In the NF-pNET patients recruited in the clinical trial, the elevated Cg A value was moreover influenced by hepatic tumor load.

**Figure 1:**
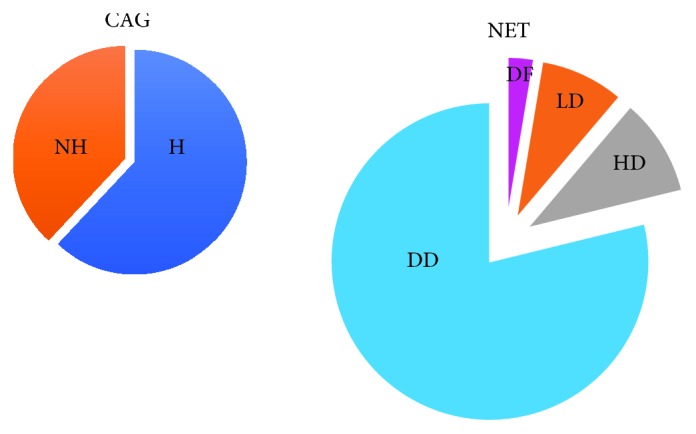
Distribution of Cg A in patients with NET and chronic atrophic gastritis (CAG). In the NET group, higher Cg A levels are observed in patients with diffuse disease (DD) compared with patients with local (LD) or hepatic (HD) disease. In the disease-free patients (DF), Cg A levels were the lowest of all. In the CAG group, higher Cg A levels are observed in patients with hyperplasia (H) of ECL cells, compared with patients without hyperplasia [66].

Campana et al. evaluated Cg A plasma levels in patients with several phenotypes of endocrine tumor and at different stages of the disease, comparing with healthy participants and patients who have chronic atrophic gastritis (CAG), with and without enterochromaffin cell-like (ECL) hyperplasia while Arnold et al. evaluated Cg A distribution in patients with NET and chronic atrophic gastritis (Figure 1) [65, 66].

In this study, higher Cg A levels are observed in cases of endocrine tumors than in those of healthy subjects, obtaining a specificity of 95.8% and a sensitivity of 85.3%, using a cutoff range of 18-19 U/L.

The patients with localized tumor showed higher levels of Cg A than CAG patients, and identifying a cutoff range of 53-54 U/L, the sensitivity was 66.5% and specificity 71.4%.

Obviously, patients with CAG showed a marker concentration higher than that of healthy participants.

These authors also noted that Cg A levels were progressively higher in the presence of advanced disease. This increase was statistically significant comparing patients with localized and diffuse disease. For this one, the used cutoff range was 281-282 U/L with sensitivity of 71.1% and specificity 78.8%.

Comparing also patients with endocrine tumors with those without tumors, they have identified a cutoff range of 31-32 U/L with sensitivity of 75.3% and specificity 84.2%.

In this analysis, a significant increase of Cg A concentration was observed in patients with GEP-NET, but a significant difference in Cg A levels was not observed in relation to the primary localization of the GEP-NET.

However, not all NET types are characterized by a correlation between Cg A elevation and extent of disease. Elevated chromogranin A is typical in gastrinoma even without metastases in the liver [71].

Instead, Koenig et al. conducted a study analyzing their GEP-NEN database and focusing only on patients with primary tumor localization in the colon and rectum [64].

The majority of tumors were well differentiated, and more than 64% of the patients exhibited metastases at diagnosis (liver and lymph nodes), especially since they were detectable in G2 and poorly differentiated G3 neoplasms.

The study demonstrated there is no correlation of plasma level of Cg A and tumor progression in patients suffering from colorectal NEN during follow-up [64].

#### 2.2.2. Type I Biomarker

“Type I” biomarkers capture the effects of an intervention in accordance with the mechanism of drug action; even though the mechanism might not be known to be associated with a clinical outcome, it still reflects interventional effects.

In assessing NET therapy, a reduction of higher than 50% or at least higher than 25% of a circulating tumor marker is considered to represent a significant effect [72–74].

The evidence to support Cg A as such a marker of therapeutic efficacy is modest and controversial.

In a study enrolling patients with progressive, metastatic NETs, including GEP-NETs, conducted by Faiss et al., the therapy with lanreotide 1 mg three times daily or interferon-*α* (INF-*α*) 5 × 10^6^ U three times weekly or lanreotide 1 mg three times daily + INF-*α* 5 × 10^6^ U three times weekly had not given significant Cg A reduction among treatment groups and the biochemical response did not correlate with inhibition of tumor growth [75].

In patients with metastatic, well-differentiated GEP-NETs, examined by Arnold et al., sudden rapid increase in Cg A to >1000 U/L was associated with increased hepatic tumor burden and rapid disease progression [66].

In a retrospective study of patients with NET, Cg A levels were the first indication of the recurrence disease after radical surgery [76]. The authors argued that its periodic measurement may be useful in detecting recurrence of the neoplasm.

Another study, conducted by Bajetta et al., showed that progression of the disease correlated with elevated Cg A concentrations in 83% of patients with GEP-NETs of different sites and in 100% of cases in patients with liver metastasis progression [77].

Brizzi et al. enrolled twenty-nine patients with locally advanced or metastatic NETs, including GEP-NETs, with radiologic confirmation of progressive disease. The treatment with octreotide LAR and 5-fluorouracil (5-FU) resulted in increased Cg A > 25% in two of twenty-five evaluable patients, stable disease in eleven patients, Cg A decrease ≥ 50% in eight of them, and complete response in four patients [78].

Vezzosi et al. enrolled 46 patients with metastatic well-differentiated GEP-NETs in a prospective study with follow-up. In 16/32 patients (50%), an increase of Cg A ≥ 25% is associated with tumor stable disease (SD) or tumor partial response (PR) (sec. *Response Evaluation Criteria In Solid Tumors*, RECIST criteria). This study does not validate Cg A as a surrogate marker of tumor progression according to RECIST criteria [79].

As somatostatin analogs decrease Cg A synthesis and secretion, the decreased blood measurements likely reflect antisecretory rather than antiproliferative effects. It is unclear whether inhibition of a secretory marker can be an effective clinical marker of cell proliferation (tumor progression).

Jacobs et al. conducted a study in which patients with metastatic, well-differentiated NETs were treated with everolimus 5 mg or 10 mg daily plus octreotide LAR 30 mg every 28 days. An early Cg A response (≥30% decrease from baseline at week 4) was observed in 77% of patients with PR, in 46% of patients with SD/progressive disease (PD), and also in 83% of carcinoid patients with tumor PD, in 38% of carcinoid patients with tumor SD/PD, and in 71% and 55% of islet-cell NET patients with tumor PR and SD/PD [80].

In the RADIANT-1 study, the Cg A response to the treatment was evaluated in addition to the baseline biomarker assessment [81]. Cg A response to everolimus occurred in 51% of patients, and an early Cg A response to the drug was observed in 47% of patients with elevated baseline levels. As a potential predictor of treatment outcomes, patients with early Cg A response had RECIST partial response. In patients with Cg A levels greater than the upper limit of the normal, measurable tumor reduction occurred in 87.1%.

In conclusion, an early Cg A response can be useful as a potential predictor of treatment outcome in patients with advanced pNET treated with everolimus.

**Figure 2:**
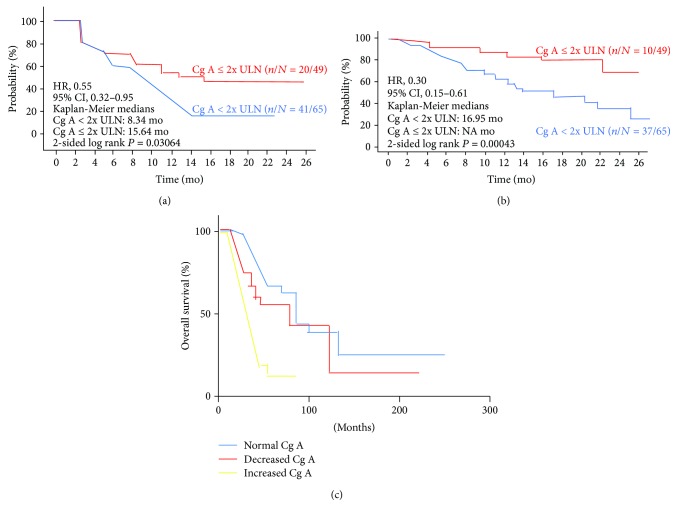
Early Cg A response as potential predictor of treatment outcome. In the RADIANT-1 study, patients with early Cg A response experience longer median progression-free survival (13.31 months) (a), whereas median overall survival is 24.90 months (b) [82]. A study in patients with NF-pNETs shows that a reduction of Cg A concentration is correlated with better prognosis in this group of patients (c) [71].

According to the study conducted by Yang et al., the therapeutic response of NF-pNETs consisted of a reduction of Cg A concentration, associated with PR and complete response in patients with this type of cancer. Contrarily, patients regarded as having progression disease or relapse had increased Cg A levels (Figure 2) [70].

Therefore, a sudden and rapid increase in serum Cg A in patients with metastatic disease should be considered as an indicator of tumor progression.

Conversely, Cg A is a very poor plasma marker for follow-up patients suffering from colorectal NENs [64].

**Figure 3:**
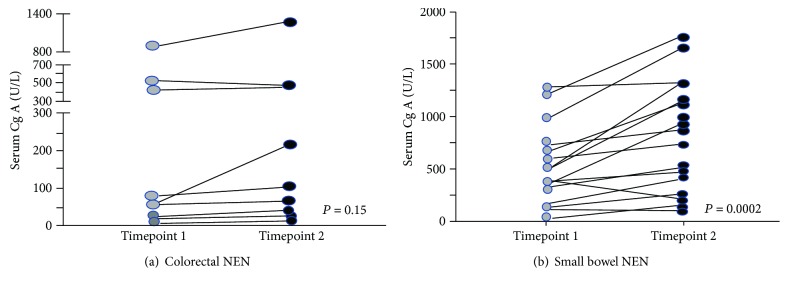
Plasma Cg A in patients with confirmed progression of colorectal NENs. Baseline levels and concentration of Cg A during follow-up of patients with metastatic colorectal NENs is no different (a) as observed during small bowel neuroendocrine tumor progression (b) [65].

Even after confirmed progression of hepatic disease, plasma levels of Cg A were not slightly elevated (Figure 3).

#### 2.2.3. Type II Biomarker

“Type II” biomarkers are considered as surrogate end points because a change in the marker predicts clinical benefit.

In a retrospective study of a mixed cohort of NETs, an early Cg A decrease after treatment was positively correlated with survival rate [82].

In patients with nonfunctioning GEP-NETs, located in the pancreas, midgut (small intestine and appendix), hindgut (large intestine, rectum, anal canal, and anus), or of unknown origin, Cg A levels are reduced with respect to baseline in the presence of treatment with lanreotide autogel at the dose of 120 mg (LAN), whereas in placebo patients, Cg A concentration increased from baseline [83]. Progression-free survival (PFS) is also significantly longer for patients receiving LAN; therefore, an increase of biomarker levels leads to a reduction in PFS.

In the prospective phase II study, patients with advanced low- to intermediate-grade NETs were enrolled to receive everolimus [4]. The trial showed that median PFS for patients with elevated baseline Cg A was 8.34 months and median overall survival (OS) was 16.95 months [81]. In addition, the data suggest that in patients with normal Cg A/elevated neuron-specific enolase (NSE), median PFS was shorter than in those with elevated Cg A/normal NSE. Median OS was worse when baseline Cg A and NSE were elevated compared with normal baseline Cg A and NSE. Early Cg A response was associated with longer median PFS (13.31 months), and the same goes for OS (24.90 months).

Normalization or ≥30% decrease of Cg A levels with everolimus monotherapy also significantly increased median PFS and OS. In conclusion, this study showed that elevated baseline Cg A levels significantly reduced OS in patients with advanced pNETs.

In pNET patients, enrolling in the RADIANT-2 trial, baseline elevated Cg A levels correlated with reduced PFS, whereas early Cg A responses were associated with significantly increased PFS, regardless of treatment [84].

In patients with low- or intermediate-grade advanced pNETs, receiving everolimus or placebo, elevated baseline Cg A determined a decrease of PFS, but the inhibitor of mammalian target of rapamycin improved progression-free survival regardless of baseline Cg A.

However, higher baseline levels of Cg A were associated with shorter PFS, showing that this marker was a poor prognostic factor of survival in patients with pNETs [85, 86].

In a study conducted by Cives et al., normal baseline Cg A correlated with median PFS of 18.5 months and elevated baseline Cg A levels, with subsequent normalization or reduction ≥ 50% within three months from the initiation of the treatment, were associated with a median PFS of 11.8 months, whereas the median PFS was 5.7 months for late and nonresponders [87].

*(1) Prognostic Role*. A prognostic role of Cg A in patients with GEP-NETs is described in the literature; in effect, high levels should correlate with shorter survival.

Retrospective studies have noted an association between Cg A levels and time survival in patients with metastatic GEP-NETs [66], pNETs [88], and nonfunctioning pNETs [89].

According Janson et al., a concentration of Cg A > 5000 mg/L is an independent predictor of shorter survival in patients with midgut carcinoids [69]; in fact, patients with Cg A less than 5000 mg/L have longer median survival (57 months) compared to patients with higher marker values (33 months).

At diagnosis of GEP-NET patients, an indicative predictor of shorter survival is a Cg A concentration in excess of three times the upper limit of the normal [88].

Modlin et al. report that tumor location and the concentration of chromogranin A do not always affect patient survival, because it is not true that the marker correlates positively with diminished survival [90].

To investigate the prognostic value for OS of Cg A in patients suffering from NF-pNETs, subjects with elevated concentration of the marker showed a lower OS than those with normal or decreased Cg A levels during the follow-up [70].

Koenig et al. showed that Cg A plasma levels are not sufficient to predict overall survival in patients with NENs of the colon and rectum [64].

## 3. Conclusions

Chromogranin A is a widely used biomarker for the assessment of neuroendocrine neoplasms, especially for the diagnosis and management of those of gastroenteropancreatic origin.

Generally, the sensitivity of this nonspecific marker test varies in different neoplasms, ranging from 10% to 100% and its specificity is from 68% to 100% [11].

The sensitivity of Cg A in the diagnosis of NETs depends on the degree of neoplasm differentiation, on the primary site and on the extent of the disease.

However, it is important to stress that the sensitivity and specificity to measurement of Cg A differ between the used commercial kits.

Because chromogranin A concentration correlates with the secretory activity of functioning tumors, a reduction of marker levels may occur during treatment with antiproliferative somatostatin analogue reflecting the inhibition of the secretory activity of the tumor rather than an antitumor effect [84].

Cg A levels cannot be used to diagnose or follow up the wide majority of patients with NENs of the colon and rectum, considering that Cg A is rarely elevated in this kind of patients, does not reflect tumor burden, and does not predict survival in these subjects [64].

Most likely, this occurs because Ec cells, expressing large amounts of Cg A, are much less frequent in the colon than in the small bowel.

Conversely, L cells, expressing small amounts of Cg A, occur mostly in the large intestinum and increase along the colon.

Several clinical trials show how higher baseline levels of Cg A are associated with shorter PFS and how an early response (a 30% or greater decrease from baseline or normalization after 4 weeks of treatment) correlates with longer PFS (13.3 mo versus 7.5 mo; HR = 0.25; *P* < 0.001) and longer OS (24.9 mo versus 12.7 mo; HR = 0.4; *P* = 0.01) [81].

In the phase III RADIANT-2 study, it has been confirmed that early decrease of Cg A concentration can represent a marker of PFS in patients on everolimus [91].

Other clinical trials have indicated the Cg A levels as a prognostic factor, including a prospective, double-blind, randomized, multicenter, phase III study (RADIANT III), which confirmed that elevated Cg A is a poor prognostic factor of survival in patients suffering from pNETs [92].

There are no comparable data for patients with GEP-NETs in treatment with sunitinib right now.

The measurement of chromogranin A should not be recommended in our daily clinical practice and could be used with caution because it has also been implicated in various benign and malignant diseases.

Cg A concentration is also a useful diagnostic biomarker in NF-pNETs and it correlates with the degree of differentiation, primary diameter of NF-pNET, and hepatic metastatic progression [70].

The assessment of circulating Cg A levels can be used as a diagnostic tool for the prediction of NETs in different treatment periods because it occurs a downward trend in the case of complete response.

Although limitations exist, Cg A levels and their change during specific therapies are prognostic, while diagnostic accuracy for GEP-NETs would seem to be higher for functioning versus nonfunctioning, well versus poorly differentiated, and metastatic versus locoregional disease.

In several clinical trials recruiting patients suffering from thoracic neuroendocrine tumors (tNETs), the tumor grade, treatment method, and Cg A status show significant correlation with survival [93].

Poor prognostic factors include not only a high grade of tNETs and incomplete resection but also a Cg A-positive status.

It is important to stress that generally the Cg A levels of patients with tNETs are significantly lower than the ones found in GEP-NETs.

Therefore, Cg A is also a poor predictive factor for tNETs.

It is also true that patients with lung NETs were enrolled in several clinical trials described above.

The results of the multivariate analysis show that baseline serum Cg A levels more than 10 times the upper limit of the normal are predictive factors of a shorter time to radiological progression and of a poor response to treatment [94].

In a phase III study (RADIANT-2), 15% of the NETs receiving treatment regimen including everolimus plus octreotide LAR occurred in the lung and a therapeutic efficacy correlated with greater reductions in serum chromogranin A [95].

Measurement of Cg A should be used to evaluate response to therapy or disease progression after all, rather than early diagnosis or recurrence.

## Figures and Tables

**Table 1:** The granin family and biological functions mediated by Cg A-derived peptides.

Granin proteins	Cg A-derived peptides	Cg A-peptide-mediated biologic function
Chromogranin A (Cg A) Chromogranin B (CgB) Secretogranin (Sg) II (CgC) Sg III (1B1075) Sg IV (HISL-19) Sg V (7B2) Sg VI (NESP55) VGF (Sg VII) proSAAS (SgVIII)	Vasostatin-1 (VST I: hCgA1–76) Vasostatin-2 (VST II: hCgA1–115) Pancreastatin (PST: hCgA357–428) Catestatin (CST: hCgA352–372) Parastatin (PARA: pCgA347–419)	Regulation of glucose balance Regulation of lipid metabolism Regulation of cardiovascular system Regulation of neurotrasmitter release Regulation of the immune system Regulation of parathormone secretion

**Table 2:** Causes of Cg A elevation unrelated to NETs.

Nononcological conditions		Nonendocrine oncological disease
Benign diseases	Iatrogenic conditions	
Gastrointestinal diseases Chronic atrophic gastritis Helicobacter pylori infection Pancreatitis Chronic hepatitis Liver cirrhosis Irritable bowel Inflammatory bowel disease Cardiovascular diseases Arterial hypertension Cardiac insufficiency Acute coronary syndrome Giant cell arteritis Renal diseases Renal insufficiency Inflammatory diseases Chronic bronchitis Obstructive pulmonary diseases Systemic rheumatoid arthritis Endocrine disease Hyperthyroidism Hyperparathyroidism	Proton pump inhibitors Histamine 2 receptor antagonists Serotonin reuptake inhibitors	Gastric carcinoma Pancreatic carcinoma Hepatocellular carcinoma Pancreatic carcinoma Breast carcinoma Ovarian carcinoma Prostate carcinoma Neuroblastoma Small cell lung cancer

## Abbreviations

NETs: Neuroendocrine tumors
NENs: Neuroendocrine neoplasms
NEC: Neuroendocrine carcinoma
MANEC: Mixed adenoneuroendocrine carcinoma
HPFs: High-power fields
GEP-NETs: Gastroenteropancreatic neuroendocrine tumors
Cg A: Chromogranin A
SCLC: Small cell lung cancer
CgB: Chromogranin B
Sg: Secretogranin
VST I: Vasostatin-1
VST II: Vasostatin-2
PST: Pancreastatin
CST: Catestatin
RIA: Radioimmunoassay
ELISA: Enzyme-linked immunosorbent assay
IRMA: Immunoradiometric assay
NSCLC: Non-small-cell lung cancer
TGN: Trans-Golgi network
DCGs: Dense-core secretory granules
CGs: Chromaffin granules
PC: Prohormone convertase family
Cg B: Chromogranin B
VEGF: Vascular endothelial growth factor
NIH: The National Institutes of Health
PPI administration: Proton pump inhibitor administration
BNP: Brain natriuretic peptide
NYHA scale: The New York Heart Association scale
MEN-1: Multiple endocrine neoplasia type 1
pNET: Pancreatic neuroendocrine tumor
CAG: Chronic atrophic gastritis
ECL: Enterochromaffin cell-like hyperplasia
INF: Interferon-*α*
5-FU: 5-fluorouracil
SD: Tumor stable disease
PR: Tumor partial response
PD: Progressive disease
RECIST: Response Evaluation Criteria in Solid Tumors
PFS: Progression-free survival
OS: Overall survival
NSE: Neuron-specific enolase.
